# Study on Antibacterial Activity of the Bark of *Garcinia lanceifolia* Roxb.

**DOI:** 10.1155/2014/784579

**Published:** 2014-10-29

**Authors:** Nilutpal Sharma Bora, Bibhuti Bhusan Kakoti, Barnali Gogoi

**Affiliations:** Department of Pharmaceutical Sciences, Dibrugarh University, Dibrugarh, Assam 786004, India

## Abstract

*Garcinia lanceifolia* Roxb. is an important and endemic medicinal plant of Assam which has been used by various ethnic communities of Northeast India to treat various disorders like dysentery, dyspepsia, and biliousness. The plant is considered to be containing much medicinal value and is also eaten raw or made into pickles by the local people. Our present study has been focused on the evaluation of the antibacterial activity of the methanolic extract of the bark of *Garcinia lanceifolia* which may lead us to a scientific evidence of the use of this plant in cases of dysentery and diarrhoea.

## 1. Introduction

*Garcinia lanceifolia* commonly known as “Rupahi-thekera” (Assamese), “Pelh” (Mizo), “Rupohi tekera” (Mising), belonging to the family Clusiaceae, is an important and endemic medicinal plant found in Assam. The plant is a handsome, small, evergreen tree. It is glabrous and grows up to a height of 12 feet under the dense shade of other trees. The leaves are about 6–12.5 × 2-3, lanceolate, long acuminate, and fleshy when green. The lateral nerves are about 8–10 on either side of the midrib which meets close to the margin. Inflorescence are polygamous, tetramerous consisting of male and hermaphrodite flowers. Male flowers are 1-2, terminal, with thick sepals, oblong fleshy with smaller petals, oblique stamens about 40 in number arranged in a glabrous mass which contains four celled anthers. The hermaphrodite flowers are terminal or axillary and larger than male flowers. Its staminoids are arranged in 4 bundles of 4-5 each; ovary ovoid, has 6–8 stigmatic rays and are glandular. Fruits are the size of small palm, ovoid, orange-yellow, and 6–8 seeded. It flowers annually between February and March while the fruiting occurs between June to July [1]. It was found previously in the evergreen forests of Assam and Meghalaya extensively; in present day it is facing the danger of extinction in nature and is often cultivated at homestead [2].

Ripe fruits are eaten raw or dried and are considered to be effective in cases of diarrhoea. The fruits and gum resin, called “gamboge,” as well as the oil and juice of the plant are used as medicine for fever, jaundice, diabetes, and urinary problems [1]. Sweet and mature fruits are eaten raw; young leaves and shoots which are slightly acidic in taste are cooked and eaten by the Karbi & Mishing tribes of Assam. The leaves of* Garcinia lanceifolia* are used as stomachic and diuretic and the fruit is used as a cure for dysentery and diarrhoea. Leaves are also cooked as vegetables and made into pickles [2]. The fruits being acidic are used to prepare juices, pickles, and other culinary preparations [3].

Plants belonging to the genus* Garcinia,* belonging to the family Clusiaceae, are known to contain bioactive compounds such as xanthones, biflavonoids, benzophenones, benzoquinones, and triterpenes which have antibacterial, antifungal, antioxidant, and cytotoxic effects [4]. The bark of* Garcinia lanceifolia* has also been reported to contain prominent anthelmintic potential [5]. Since the fruits and leaves of the plants have been used in cases of dysentery and diarrhoea, the plant may contain good antibacterial activity. Therefore, this study was undertaken to establish the antibacterial properties on four different species of microbes, namely,* Bacillus subtilis*,* Staphylococcus aureus*,* Pseudomonas aeruginosa*, and* Escherichia coli*. The test organisms were maintained on nutrient agar slants.

## 2. Materials and Method

### 2.1. Plant Collection and Preparation of Extract

The bark of* Garcinia lanceifolia* was collected in the month of August, 2013, from the campus of Dibrugarh University and neighboring areas of Dibrugarh, Assam, India. The plant was identified and authenticated by Dr. A. A. Mao, Botanical Survey of India, Eastern Regional Centre, Shillong vide identification number BSI/ERC/2014/Plant identification/882. The voucher specimen of the plant was deposited in the Research Lab of the Department for further references. The bark was cut into pieces and washed thoroughly with water. The leaves were then dried partially under sunlight and partially under the shade for a week. The dried bark pieces were then ground in mechanical grinder and stored in airtight containers free from moisture.

The methanolic extract of the stem bark of* Garcinia lanceifolia* (MEGL) was prepared by Continuous Hot Percolation (Soxhlet Extraction) using 1000 mL of methanol after pretreatment with petroleum ether. The extracts were concentrated by distilling out the solvent and then vacuum dried using a rotary evaporator. Preliminary phytochemical tests were carried out with all the extracts in order to evaluate for the presence of different phytochemical constituents.

### 2.2. Growth and Maintenance of Test Microorganism

The bacterium strains of* Bacillus subtilis* (*B. subtilis*),* Staphylococcus aureus* (*S. aureus*),* Pseudomonas aeruginosa* (*P. aeruginosa*), and* Escherichia coli* (*E. coli*) were maintained at 37°C in Nutrient Broth until the preparation of inoculum.

### 2.3. Preparation of the Inoculum

The gram positive (*Bacillus subtilis* and* Staphylococcus aureus*) and gram negative bacteria (*Escherichia coli* and* Pseudomonas aeruginosa*) were cultured in nutrient broth for 24 h. This bacterial culture was used as an inoculum for the antibacterial assay. The plating was carried out by transferring bacterial suspension (10^5^ CFU/mL) mixed with Mueller Hinton Agar (Hi-Media Laboratories Limited, Mumbai, India) to sterile petri plates and allowed to solidify.

### 2.4. Phytochemical Screening

The different extracts obtained by successive solvent extraction were tested separately for the presence of various phytoconstituents; namely, alkaloids, amino acids, carbohydrates, fats and fixed oils, flavonoids, glycosides, saponins, gums, lignins, proteins, steroids, triterpenoids, tannins, and phenolic compounds. The extensive screening was carried out to establish the phytochemical fingerprint in MEGL [6].

### 2.5. Antibacterial Activity

In vitro antibacterial activity was determined by using the agar well-diffusion method [4]. Four wells were bored into each of the solidified agar plants using a cork borer and were treated with the standard drug, Ciprofloxacin (10 *μ*g/mL): two doses of the MEGL 10 *μ*g/mL and 100 *μ*g/mL and one negative control of methanol solvent only. Each well was found to contain 75 *μ*L of the above samples. The plates were incubated at 37° for 24 h and the activity was determined by measuring the diameter of inhibition zones in mm. All the assessments were done in triplicates.

## 3. Results and Discussions

### 3.1. Phytochemical Screening

The methanolic bark extract of the bark of* Garcinia lanceifolia* is found to contain many important phytochemical constituents, namely, tannins and phenolic compounds, triterpenoids, lignins, proteins, flavonoids, glycosides, amino acids, and carbohydrates. Furthermore, it can be mentioned that when determination was carried out by using in vitro methods the content of phenolic compounds and flavonoids was found to be very high. Qualitative determination of other constituents is underway which will provide the detailed amount of the various constituents present.

### 3.2. Antibacterial Activity

**Figure 1:**
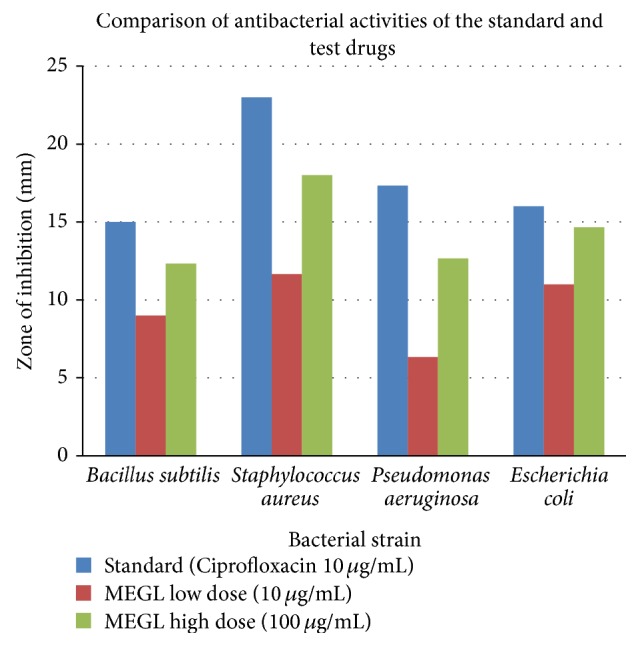
Comparison of antibacterial activities of the standard and test drugs.

As stated earlier* Garcinia lanceifolia* is an important, endemic, and endangered species of medicinal plant found in Assam and is used by the local people to treat moderate to severe cases of dysentery and diarrhoea. Therefore, in order to ascertain the antibacterial activity of this plant the present study was undertaken. The MEGL showed significant activity against gram positive bacteria as compared to the gram negative bacteria. The results have been tabulated in Table 1. The different strengths of the MEGL have been found to demonstrate appreciable antibacterial activity against various bacterial strains when compared to the standard drug Ciprofloxacin. The comparisons have been shown in Figure 1.

The most susceptible strain was found to be* Staphylococcus aureus*, whereas the least susceptible strain was found to be* Pseudomonas aeruginosa*. The antibacterial activity of MEGL was found to be higher in case of the higher dose which proved that the antibacterial activity of MEGL should increase in a dose dependent manner. The results of this study revealed the underlying fact that the antidiarrheal and antidysenteric activity of the plant of* Garcinia lanceifolia* may be attributed to antibacterial activity which was proved scientifically in this study.

## 4. Conclusion

As found from the study that the methanolic extract of the bark of the plant* Garcinia lanceifolia* has shown significant antibacterial activity against four strains of bacterium, namely,* Bacillus subtilis* (*B. subtilis*),* Staphylococcus aureus* (*S. aureus*),* Pseudomonas aeruginosa* (*P. aeruginosa*), and* Escherichia coli* (*E. coli*). The MEGL is seen to have more activity against gram positive bacteria than gram negative bacteria and in a dose dependent manner. As the doses were increased the activity is also found to increase. This study can be a basis for finding out new avenues in the field of antibacterial and can help in the elucidation of the actual mechanism of action in the antibacterial action of this plant. Further studies have also been undertaken for isolating new compounds from the extract so that it can lead to the development of newer and safer antibacterial drugs. The methanolic extract has been subjected through fractionation using column and flash chromatography and has been isolated into fractions of chloroform, ethyl acetate, acetonitrile, and n-butanol. Since this preliminary study showed the presence of antibacterial activity, further studies on these fractions will be undertaken so as to determine which constituent(s) was responsible for the aforesaid activity.

## Figures and Tables

**Table 1:** Antibacterial activity of the methanolic extract of the bark of *Garcinia lanceifolia*.

	Zone of inhibition (in mm)			
Bacterial strain	Control (methanol)	Standard	MEGL low dose	MEGL high dose
		(Ciprofloxacin 10 *μ*g/mL)	(10 *μ*g/mL)	(100 *μ*g/mL)
*Bacillus subtilis *	—	15.00 ± 0.574	9.00 ± 0.577	12.33 ± 0.333
*Staphylococcus aureus *	—	23.00 ± 1.155	11.66 ± 0.666	18.00 ± 0.577
*Pseudomonas aeruginosa *	—	17.33 ± 0.667	6.33 ± 0.333	12.66 ± 0.666
*Escherichia coli *	—	16.00 ± 1.000	11.00 ± 0.577	14.66 ± 0.881

## Abbreviations

MEGL: Methanolic extract of the bark of* Garcinia lanceifolia.*
